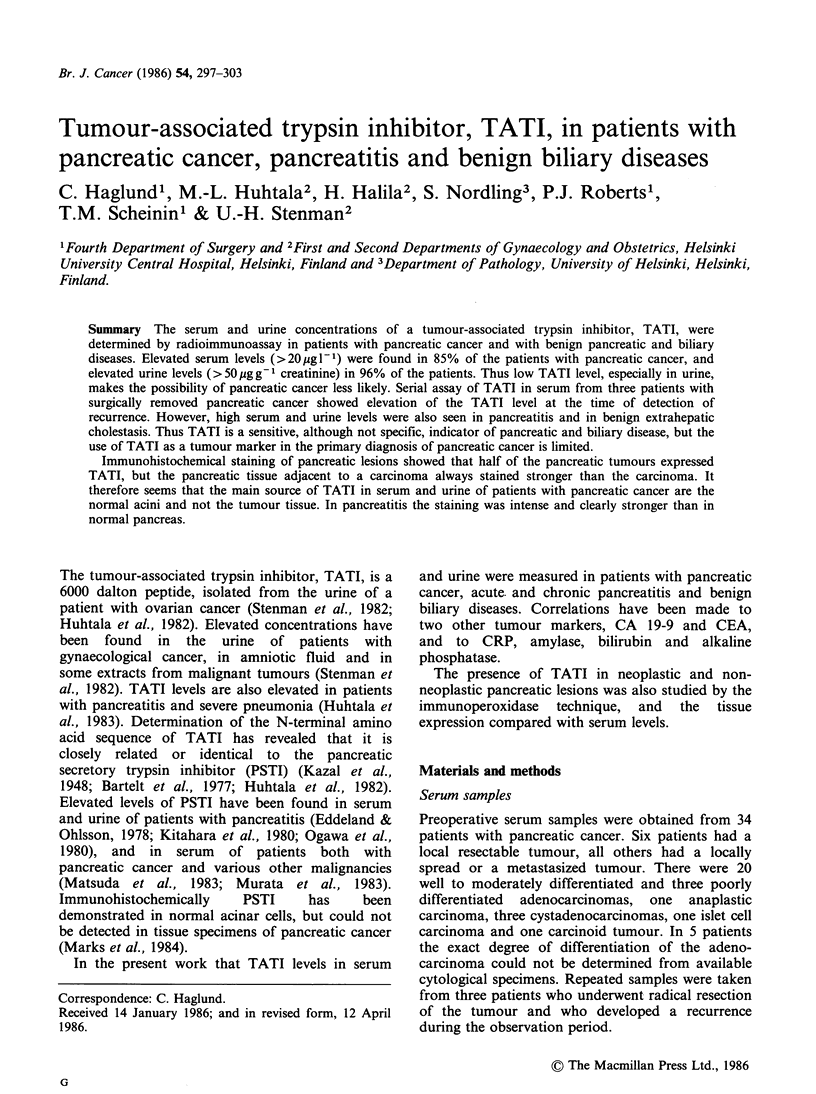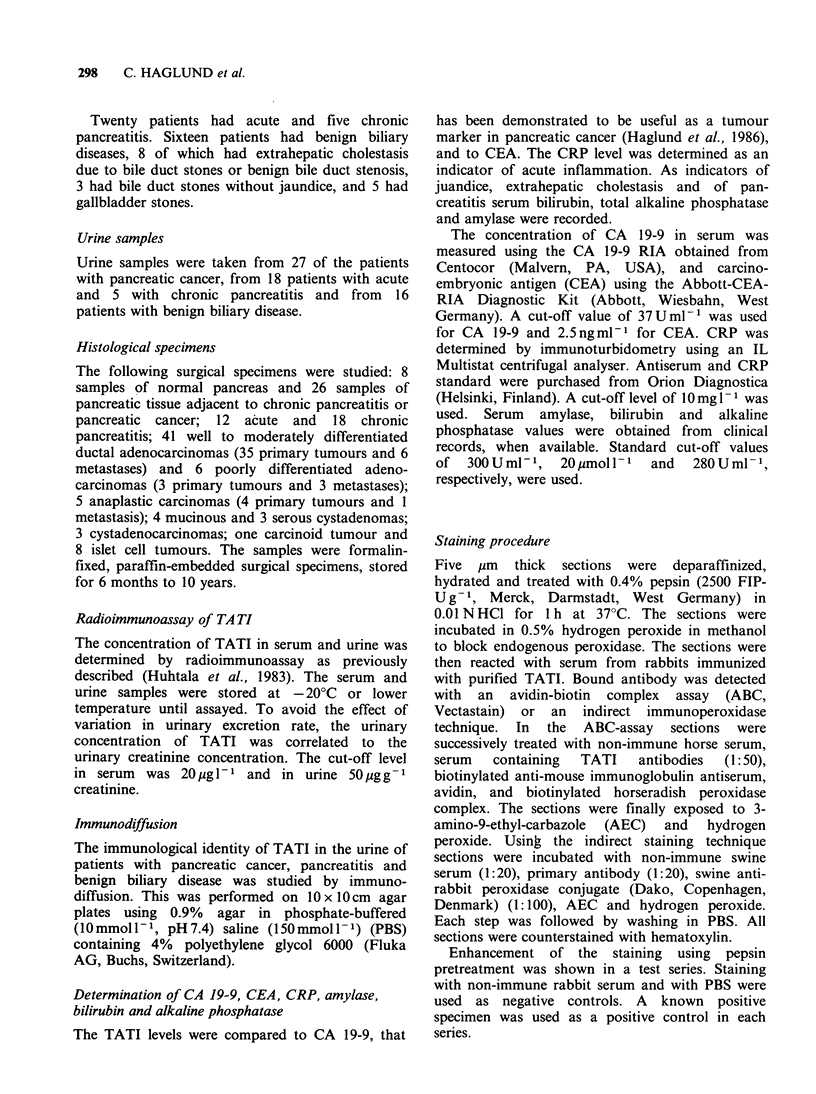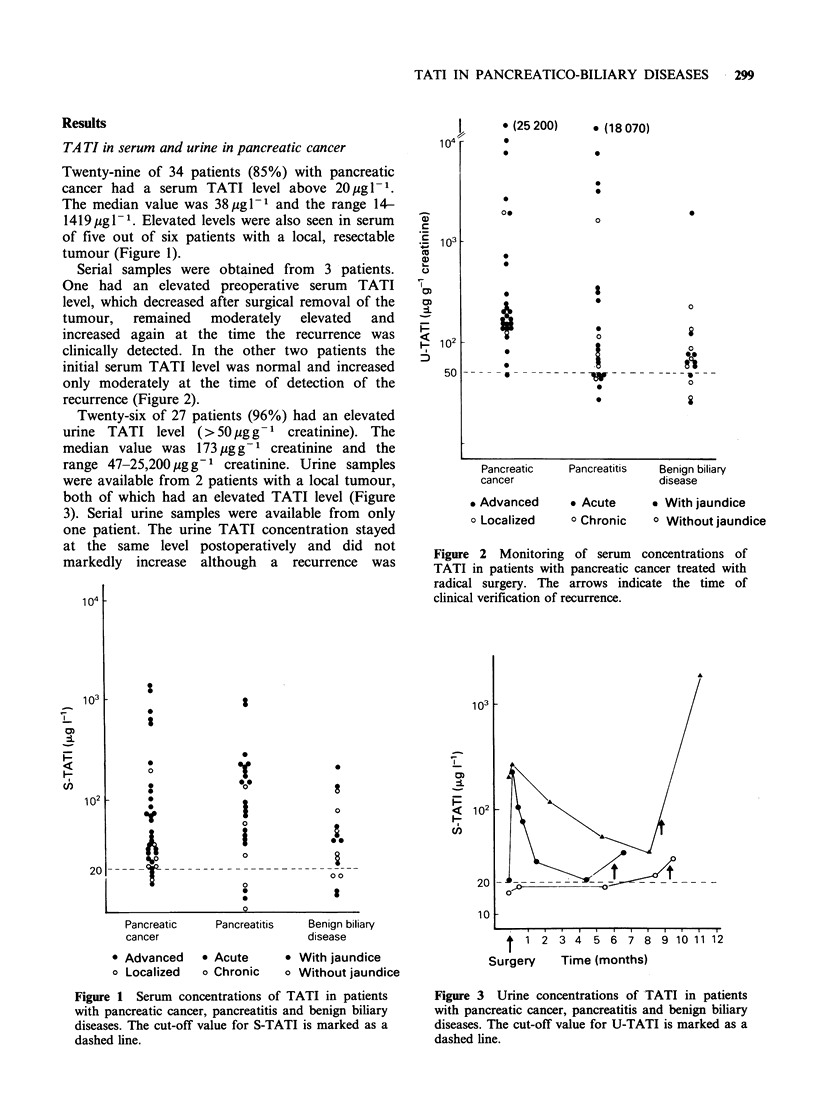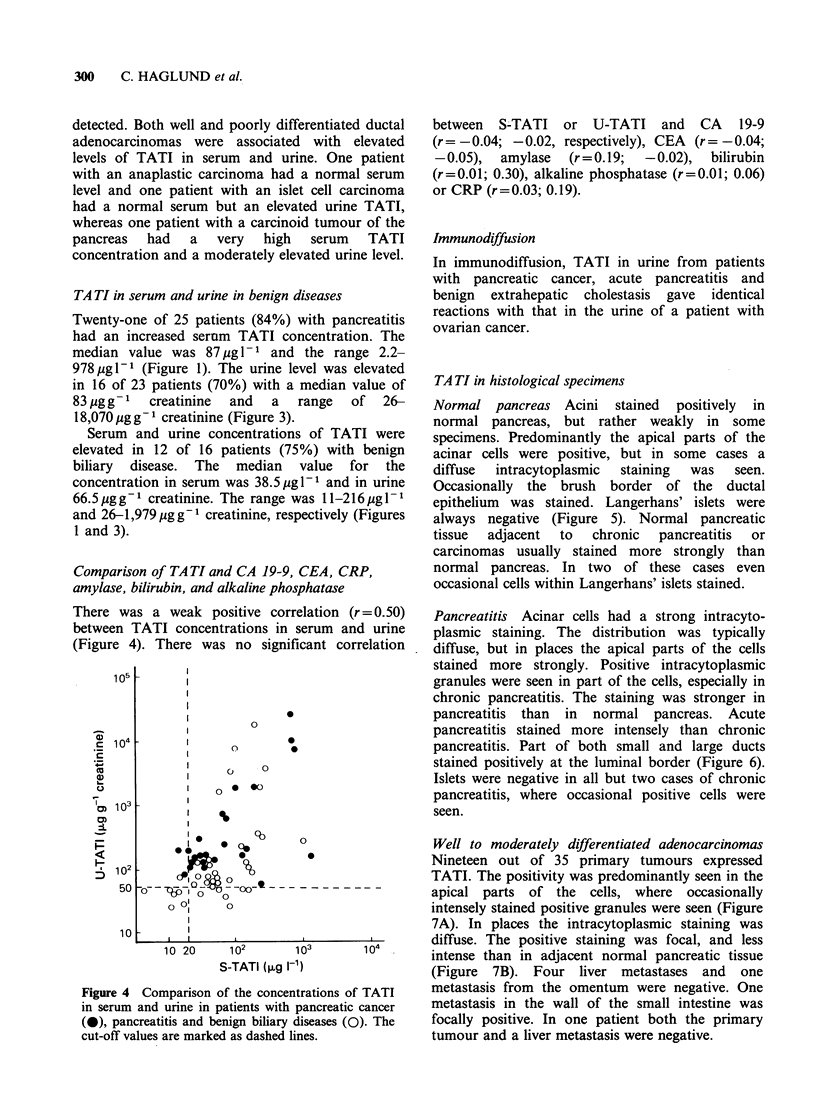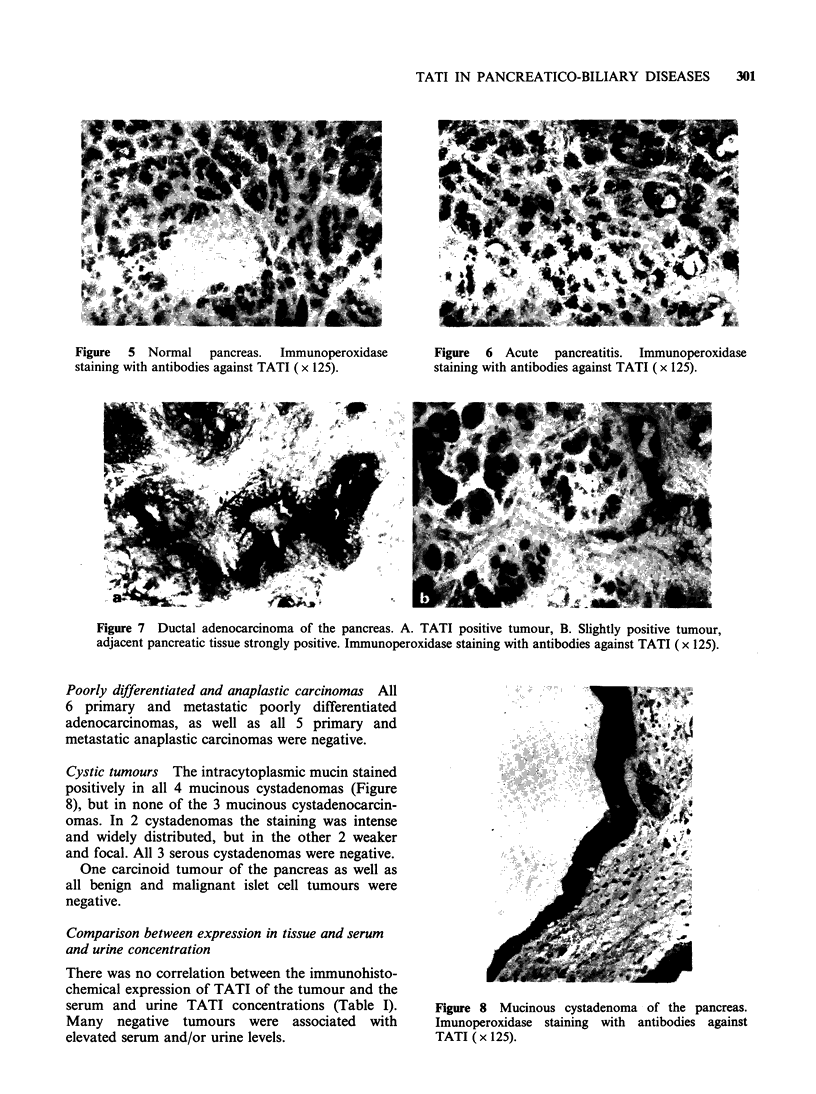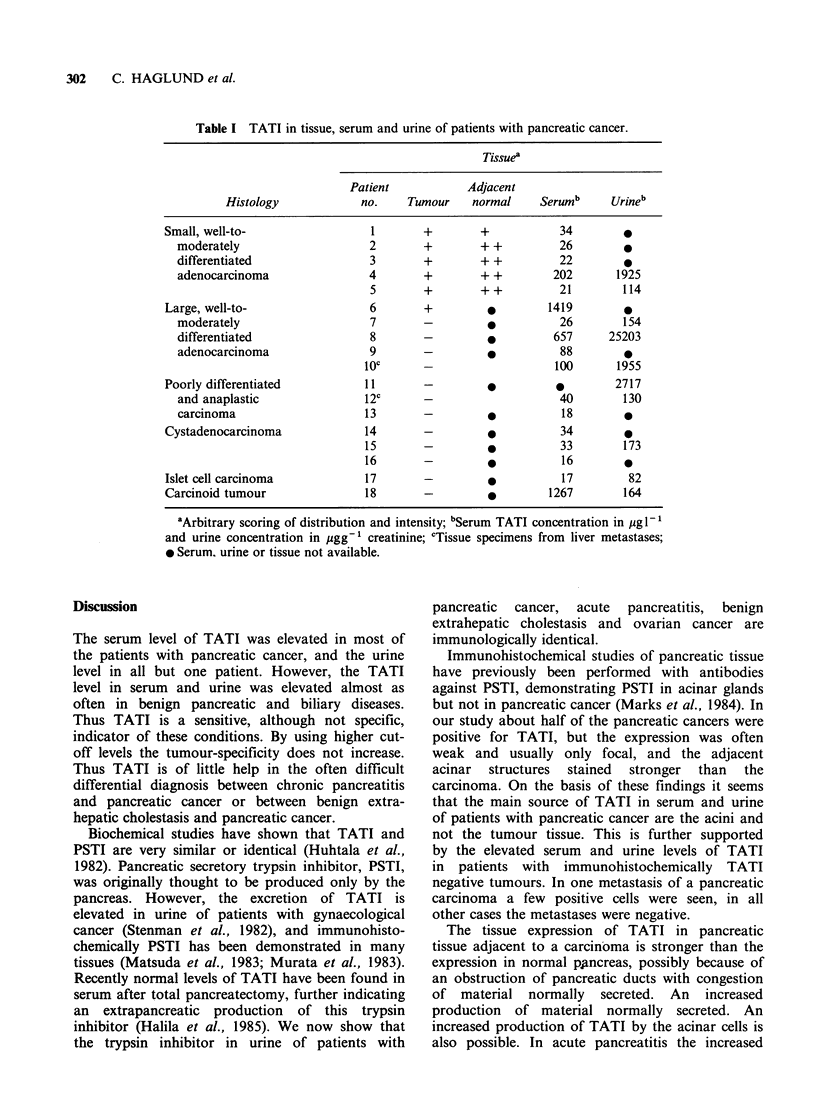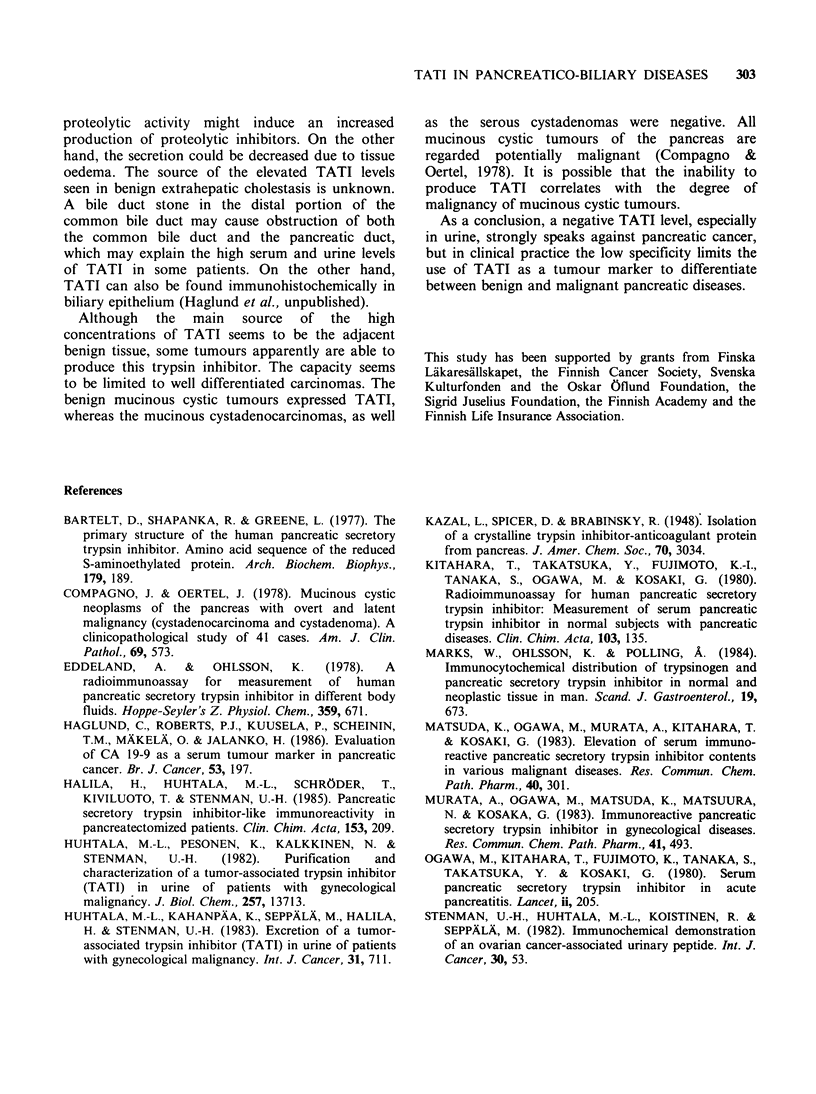# Tumour-associated trypsin inhibitor, TATI, in patients with pancreatic cancer, pancreatitis and benign biliary diseases.

**DOI:** 10.1038/bjc.1986.176

**Published:** 1986-08

**Authors:** C. Haglund, M. L. Huhtala, H. Halila, S. Nordling, P. J. Roberts, T. M. Scheinin, U. H. Stenman

## Abstract

**Images:**


*Br. J. Cancer* (1986) **54**, 297–303

### Tumour-associated trypsin inhibitor, TATI, in patients with pancreatic cancer, pancreatitis and benign biliary diseases

C. Haglund[1], M.-L. Huhtala[2], H. Halila[2], S. Nordling[3], P.J. Roberts[1], T.M. Scheinin[1] & U.-H. Stenman[2]

[1]*Fourth Department of Surgery and* [2]*First and Second Departments of Gynaecology and Obstetrics, Helsinki University Central Hospital, Helsinki, Finland and* [3]*Department of Pathology, University of Helsinki, Helsinki, Finland.*

**Summary** The serum and urine concentrations of a tumour-associated trypsin inhibitor, TATI, were determined by radioimmunoassay in patients with pancreatic cancer and with benign pancreatic and biliary diseases. Elevated serum levels ($>20\,\mu g\,l^{-1}$) were found in 85% of the patients with pancreatic cancer, and elevated urine levels ($>50\,\mu g\,g^{-1}$ creatinine) in 96% of the patients. Thus low TATI level, especially in urine, makes the possibility of pancreatic cancer less likely. Serial assay of TATI in serum from three patients with surgically removed pancreatic cancer showed elevation of the TATI level at the time of detection of recurrence. However, high serum and urine levels were also seen in pancreatitis and in benign extrahepatic cholestasis. Thus TATI is a sensitive, although not specific, indicator of pancreatic and biliary disease, but the use of TATI as a tumour marker in the primary diagnosis of pancreatic cancer is limited.

Immunohistochemical staining of pancreatic lesions showed that half of the pancreatic tumours expressed TATI, but the pancreatic tissue adjacent to a carcinoma always stained stronger than the carcinoma. It therefore seems that the main source of TATI in serum and urine of patients with pancreatic cancer are the normal acini and not the tumour tissue. In pancreatitis the staining was intense and clearly stronger than in normal pancreas.

The tumour-associated trypsin inhibitor, TATI, is a 6000 dalton peptide, isolated from the urine of a patient with ovarian cancer (Stenman *et al.*, 1982; Huhtala *et al.*, 1982). Elevated concentrations have been found in the urine of patients with gynaecological cancer, in amniotic fluid and in some extracts from malignant tumours (Stenman *et al.*, 1982). TATI levels are also elevated in patients with pancreatitis and severe pneumonia (Huhtala *et al.*, 1983). Determination of the N-terminal amino acid sequence of TATI has revealed that it is closely related or identical to the pancreatic secretory trypsin inhibitor (PSTI) (Kazal *et al.*, 1948; Bartelt *et al.*, 1977; Huhtala *et al.*, 1982). Elevated levels of PSTI have been found in serum and urine of patients with pancreatitis (Eddeland & Ohlsson, 1978; Kitahara *et al.*, 1980; Ogawa *et al.*, 1980), and in serum of patients both with pancreatic cancer and various other malignancies (Matsuda *et al.*, 1983; Murata *et al.*, 1983). Immunohistochemically PSTI has been demonstrated in normal acinar cells, but could not be detected in tissue specimens of pancreatic cancer (Marks *et al.*, 1984).

In the present work that TATI levels in serum and urine were measured in patients with pancreatic cancer, acute and chronic pancreatitis and benign biliary diseases. Correlations have been made to two other tumour markers, CA 19-9 and CEA, and to CRP, amylase, bilirubin and alkaline phosphatase.

The presence of TATI in neoplastic and non-neoplastic pancreatic lesions was also studied by the immunoperoxidase technique, and the tissue expression compared with serum levels.

Correspondence: C. Haglund.
Received 14 January 1986; and in revised form, 12 April 1986.

### Materials and methods

### *Serum samples*

Preoperative serum samples were obtained from 34 patients with pancreatic cancer. Six patients had a local resectable tumour, all others had a locally spread or a metastasized tumour. There were 20 well to moderately differentiated and three poorly differentiated adenocarcinomas, one anaplastic carcinoma, three cystadenocarcinomas, one islet cell carcinoma and one carcinoid tumour. In 5 patients the exact degree of differentiation of the adenocarcinoma could not be determined from available cytological specimens. Repeated samples were taken from three patients who underwent radical resection of the tumour and who developed a recurrence during the observation period.

© The Macmillan Press Ltd., 1986

Twenty patients had acute and five chronic pancreatitis. Sixteen patients had benign biliary diseases, 8 of which had extrahepatic cholestasis due to bile duct stones or benign bile duct stenosis, 3 had bile duct stones without jaundice, and 5 had gallbladder stones.

### *Urine samples*

Urine samples were taken from 27 of the patients with pancreatic cancer, from 18 patients with acute and 5 with chronic pancreatitis and from 16 patients with benign biliary disease.

### *Histological specimens*

The following surgical specimens were studied: 8 samples of normal pancreas and 26 samples of pancreatic tissue adjacent to chronic pancreatitis or pancreatic cancer; 12 acute and 18 chronic pancreatitis; 41 well to moderately differentiated ductal adenocarcinomas (35 primary tumours and 6 metastases) and 6 poorly differentiated adenocarcinomas (3 primary tumours and 3 metastases); 5 anaplastic carcinomas (4 primary tumours and 1 metastasis); 4 mucinous and 3 serous cystadenomas; 3 cystadenocarcinomas; one carcinoid tumour and 8 islet cell tumours. The samples were formalin-fixed, paraffin-embedded surgical specimens, stored for 6 months to 10 years.

### *Radioimmunoassay of TATI*

The concentration of TATI in serum and urine was determined by radioimmunoassay as previously described (Huhtala *et al.*, 1983). The serum and urine samples were stored at −20°C or lower temperature until assayed. To avoid the effect of variation in urinary excretion rate, the urinary concentration of TATI was correlated to the urinary creatinine concentration. The cut-off level in serum was $20\,\mu g\,l^{-1}$ and in urine $50\,\mu g\,g^{-1}$ creatinine.

### *Immunodiffusion*

The immunological identity of TATI in the urine of patients with pancreatic cancer, pancreatitis and benign biliary disease was studied by immunodiffusion. This was performed on 10 × 10 cm agar plates using 0.9% agar in phosphate-buffered ($10\,mmol\,l^{-1}$, pH 7.4) saline ($150\,mmol\,l^{-1}$) (PBS) containing 4% polyethylene glycol 6000 (Fluka AG, Buchs, Switzerland).

### *Determination of CA 19-9, CEA, CRP, amylase, bilirubin and alkaline phosphatase*

The TATI levels were compared to CA 19-9, that has been demonstrated to be useful as a tumour marker in pancreatic cancer (Haglund *et al.*, 1986), and to CEA. The CRP level was determined as an indicator of acute inflammation. As indicators of juandice, extrahepatic cholestasis and of pancreatitis serum bilirubin, total alkaline phosphatase and amylase were recorded.

The concentration of CA 19-9 in serum was measured using the CA 19-9 RIA obtained from Centocor (Malvern, PA, USA), and carcinoembryonic antigen (CEA) using the Abbott-CEA-RIA Diagnostic Kit (Abbott, Wiesbahn, West Germany). A cut-off value of $37\,U\,ml^{-1}$ was used for CA 19-9 and $2.5\,ng\,ml^{-1}$ for CEA. CRP was determined by immunoturbidometry using an IL Multistat centrifugal analyser. Antiserum and CRP standard were purchased from Orion Diagnostica (Helsinki, Finland). A cut-off level of $10\,mg\,l^{-1}$ was used. Serum amylase, bilirubin and alkaline phosphatase values were obtained from clinical records, when available. Standard cut-off values of $300\,U\,ml^{-1}$, $20\,\mu mol\,l^{-1}$ and $280\,U\,ml^{-1}$, respectively, were used.

### *Staining procedure*

Five $\mu m$ thick sections were deparaffinized, hydrated and treated with 0.4% pepsin (2500 FIP-$U\,g^{-1}$, Merck, Darmstadt, West Germany) in 0.01 N HCl for 1 h at 37°C. The sections were incubated in 0.5% hydrogen peroxide in methanol to block endogenous peroxidase. The sections were then reacted with serum from rabbits immunized with purified TATI. Bound antibody was detected with an avidin-biotin complex assay (ABC, Vectastain) or an indirect immunoperoxidase technique. In the ABC-assay sections were successively treated with non-immune horse serum, serum containing TATI antibodies (1:50), biotinylated anti-mouse immunoglobulin antiserum, avidin, and biotinylated horseradish peroxidase complex. The sections were finally exposed to 3-amino-9-ethyl-carbazole (AEC) and hydrogen peroxide. Using the indirect staining technique sections were incubated with non-immune swine serum (1:20), primary antibody (1:20), swine anti-rabbit peroxidase conjugate (Dako, Copenhagen, Denmark) (1:100), AEC and hydrogen peroxide. Each step was followed by washing in PBS. All sections were counterstained with hematoxylin.

Enhancement of the staining using pepsin pretreatment was shown in a test series. Staining with non-immune rabbit serum and with PBS were used as negative controls. A known positive specimen was used as a positive control in each series.

### Results

*TATI in serum and urine in pancreatic cancer*

Twenty-nine of 34 patients (85%) with pancreatic cancer had a serum TATI level above $20\,\mu g\,l^{-1}$. The median value was $38\,\mu g\,l^{-1}$ and the range 14–1419 $\mu g\,l^{-1}$. Elevated levels were also seen in serum of five out of six patients with a local, resectable tumour (Figure 1).

Serial samples were obtained from 3 patients. One had an elevated preoperative serum TATI level, which decreased after surgical removal of the tumour, remained moderately elevated and increased again at the time the recurrence was clinically detected. In the other two patients the initial serum TATI level was normal and increased only moderately at the time of detection of the recurrence (Figure 2).

Twenty-six of 27 patients (96%) had an elevated urine TATI level ($>50\,\mu g\,g^{-1}$ creatinine). The median value was $173\,\mu g\,g^{-1}$ creatinine and the range 47–25,200 $\mu g\,g^{-1}$ creatinine. Urine samples were available from 2 patients with a local tumour, both of which had an elevated TATI level (Figure 3). Serial urine samples were available from only one patient. The urine TATI concentration stayed at the same level postoperatively and did not markedly increase although a recurrence was

**Figure 1** Serum concentrations of TATI in patients with pancreatic cancer, pancreatitis and benign biliary diseases. The cut-off value for S-TATI is marked as a dashed line.

**Figure 2** Monitoring of serum concentrations of TATI in patients with pancreatic cancer treated with radical surgery. The arrows indicate the time of clinical verification of recurrence.

**Figure 3** Urine concentrations of TATI in patients with pancreatic cancer, pancreatitis and benign biliary diseases. The cut-off value for U-TATI is marked as a dashed line.

detected. Both well and poorly differentiated ductal adenocarcinomas were associated with elevated levels of TATI in serum and urine. One patient with an anaplastic carcinoma had a normal serum level and one patient with an islet cell carcinoma had a normal serum but an elevated urine TATI, whereas one patient with a carcinoid tumour of the pancreas had a very high serum TATI concentration and a moderately elevated urine level.

*TATI in serum and urine in benign diseases*

Twenty-one of 25 patients (84%) with pancreatitis had an increased serum TATI concentration. The median value was 87 $\mu g l^{-1}$ and the range 2.2–978 $\mu g l^{-1}$ (Figure 1). The urine level was elevated in 16 of 23 patients (70%) with a median value of 83 $\mu g g^{-1}$ creatinine and a range of 26–18,070 $\mu g g^{-1}$ creatinine (Figure 3).

Serum and urine concentrations of TATI were elevated in 12 of 16 patients (75%) with benign biliary disease. The median value for the concentration in serum was 38.5 $\mu g l^{-1}$ and in urine 66.5 $\mu g g^{-1}$ creatinine. The range was 11–216 $\mu g l^{-1}$ and 26–1,979 $\mu g g^{-1}$ creatinine, respectively (Figures 1 and 3).

*Comparison of TATI and CA 19-9, CEA, CRP, amylase, bilirubin, and alkaline phosphatase*

There was a weak positive correlation ($r = 0.50$) between TATI concentrations in serum and urine (Figure 4). There was no significant correlation between S-TATI or U-TATI and CA 19-9 ($r = -0.04$; $-0.02$, respectively), CEA ($r = -0.04$; $-0.05$), amylase ($r = 0.19$; $-0.02$), bilirubin ($r = 0.01$; 0.30), alkaline phosphatase ($r = 0.01$; 0.06) or CRP ($r = 0.03$; 0.19).

**Figure 4** Comparison of the concentrations of TATI in serum and urine in patients with pancreatic cancer (●), pancreatitis and benign biliary diseases (○). The cut-off values are marked as dashed lines.

*Immunodiffusion*

In immunodiffusion, TATI in urine from patients with pancreatic cancer, acute pancreatitis and benign extrahepatic cholestasis gave identical reactions with that in the urine of a patient with ovarian cancer.

*TATI in histological specimens*

*Normal pancreas* Acini stained positively in normal pancreas, but rather weakly in some specimens. Predominantly the apical parts of the acinar cells were positive, but in some cases a diffuse intracytoplasmic staining was seen. Occasionally the brush border of the ductal epithelium was stained. Langerhans' islets were always negative (Figure 5). Normal pancreatic tissue adjacent to chronic pancreatitis or carcinomas usually stained more strongly than normal pancreas. In two of these cases even occasional cells within Langerhans' islets stained.

*Pancreatitis* Acinar cells had a strong intracytoplasmic staining. The distribution was typically diffuse, but in places the apical parts of the cells stained more strongly. Positive intracytoplasmic granules were seen in part of the cells, especially in chronic pancreatitis. The staining was stronger in pancreatitis than in normal pancreas. Acute pancreatitis stained more intensely than chronic pancreatitis. Part of both small and large ducts stained positively at the luminal border (Figure 6). Islets were negative in all but two cases of chronic pancreatitis, where occasional positive cells were seen.

*Well to moderately differentiated adenocarcinomas* Nineteen out of 35 primary tumours expressed TATI. The positivity was predominantly seen in the apical parts of the cells, where occasionally intensely stained positive granules were seen (Figure 7A). In places the intracytoplasmic staining was diffuse. The positive staining was focal, and less intense than in adjacent normal pancreatic tissue (Figure 7B). Four liver metastases and one metastasis from the omentum were negative. One metastasis in the wall of the small intestine was focally positive. In one patient both the primary tumour and a liver metastasis were negative.

**Figure 5** Normal pancreas. Immunoperoxidase staining with antibodies against TATI ( × 125).

**Figure 6** Acute pancreatitis. Immunoperoxidase staining with antibodies against TATI ( × 125).

**Figure 7** Ductal adenocarcinoma of the pancreas. A. TATI positive tumour, B. Slightly positive tumour, adjacent pancreatic tissue strongly positive. Immunoperoxidase staining with antibodies against TATI ( × 125).

*Poorly differentiated and anaplastic carcinomas* All 6 primary and metastatic poorly differentiated adenocarcinomas, as well as all 5 primary and metastatic anaplastic carcinomas were negative.

*Cystic tumours* The intracytoplasmic mucin stained positively in all 4 mucinous cystadenomas (Figure 8), but in none of the 3 mucinous cystadenocarcinomas. In 2 cystadenomas the staining was intense and widely distributed, but in the other 2 weaker and focal. All 3 serous cystadenomas were negative.

One carcinoid tumour of the pancreas as well as all benign and malignant islet cell tumours were negative.

*Comparison between expression in tissue and serum and urine concentration*

There was no correlation between the immunohistochemical expression of TATI of the tumour and the serum and urine TATI concentrations (Table I). Many negative tumours were associated with elevated serum and/or urine levels.

**Figure 8** Mucinous cystadenoma of the pancreas. Imunoperoxidase staining with antibodies against TATI ( × 125).

**Table I** TATI in tissue, serum and urine of patients with pancreatic cancer.

| *Histology* | *Patient no.* | *Tissue*[a] *Tumour* | *Tissue*[a] *Adjacent normal* | *Serum*[b] | *Urine*[b] |
|---|---|---|---|---|---|
| Small, well-to- | 1 | + | + | 34 | ● |
| moderately | 2 | + | ++ | 26 | ● |
| differentiated | 3 | + | ++ | 22 | ● |
| adenocarcinoma | 4 | + | ++ | 202 | 1925 |
| | 5 | + | ++ | 21 | 114 |
| Large, well-to- | 6 | + | ● | 1419 | ● |
| moderately | 7 | − | ● | 26 | 154 |
| differentiated | 8 | − | ● | 657 | 25203 |
| adenocarcinoma | 9 | − | ● | 88 | ● |
| | 10[c] | − | | 100 | 1955 |
| Poorly differentiated | 11 | − | ● | ● | 2717 |
| and anaplastic | 12[c] | − | | 40 | 130 |
| carcinoma | 13 | − | ● | 18 | ● |
| Cystadenocarcinoma | 14 | − | ● | 34 | ● |
| | 15 | − | ● | 33 | 173 |
| | 16 | − | ● | 16 | ● |
| Islet cell carcinoma | 17 | − | ● | 17 | 82 |
| Carcinoid tumour | 18 | − | ● | 1267 | 164 |

[a]Arbitrary scoring of distribution and intensity; [b]Serum TATI concentration in $\mu g l^{-1}$ and urine concentration in $\mu g g^{-1}$ creatinine; [c]Tissue specimens from liver metastases; ● Serum. urine or tissue not available.

### Discussion

The serum level of TATI was elevated in most of the patients with pancreatic cancer, and the urine level in all but one patient. However, the TATI level in serum and urine was elevated almost as often in benign pancreatic and biliary diseases. Thus TATI is a sensitive, although not specific, indicator of these conditions. By using higher cut-off levels the tumour-specificity does not increase. Thus TATI is of little help in the often difficult differential diagnosis between chronic pancreatitis and pancreatic cancer or between benign extra-hepatic cholestasis and pancreatic cancer.

Biochemical studies have shown that TATI and PSTI are very similar or identical (Huhtala *et al.*, 1982). Pancreatic secretory trypsin inhibitor, PSTI, was originally thought to be produced only by the pancreas. However, the excretion of TATI is elevated in urine of patients with gynaecological cancer (Stenman *et al.*, 1982), and immunohisto-chemically PSTI has been demonstrated in many tissues (Matsuda *et al.*, 1983; Murata *et al.*, 1983). Recently normal levels of TATI have been found in serum after total pancreatectomy, further indicating an extrapancreatic production of this trypsin inhibitor (Halila *et al.*, 1985). We now show that the trypsin inhibitor in urine of patients with pancreatic cancer, acute pancreatitis, benign extrahepatic cholestasis and ovarian cancer are immunologically identical.

Immunohistochemical studies of pancreatic tissue have previously been performed with antibodies against PSTI, demonstrating PSTI in acinar glands but not in pancreatic cancer (Marks *et al.*, 1984). In our study about half of the pancreatic cancers were positive for TATI, but the expression was often weak and usually only focal, and the adjacent acinar structures stained stronger than the carcinoma. On the basis of these findings it seems that the main source of TATI in serum and urine of patients with pancreatic cancer are the acini and not the tumour tissue. This is further supported by the elevated serum and urine levels of TATI in patients with immunohistochemically TATI negative tumours. In one metastasis of a pancreatic carcinoma a few positive cells were seen, in all other cases the metastases were negative.

The tissue expression of TATI in pancreatic tissue adjacent to a carcinoma is stronger than the expression in normal pancreas, possibly because of an obstruction of pancreatic ducts with congestion of material normally secreted. An increased production of material normally secreted. An increased production of TATI by the acinar cells is also possible. In acute pancreatitis the increased proteolytic activity might induce an increased production of proteolytic inhibitors. On the other hand, the secretion could be decreased due to tissue oedema. The source of the elevated TATI levels seen in benign extrahepatic cholestasis is unknown. A bile duct stone in the distal portion of the common bile duct may cause obstruction of both the common bile duct and the pancreatic duct, which may explain the high serum and urine levels of TATI in some patients. On the other hand, TATI can also be found immunohistochemically in biliary epithelium (Haglund *et al.*, unpublished).

Although the main source of the high concentrations of TATI seems to be the adjacent benign tissue, some tumours apparently are able to produce this trypsin inhibitor. The capacity seems to be limited to well differentiated carcinomas. The benign mucinous cystic tumours expressed TATI, whereas the mucinous cystadenocarcinomas, as well as the serous cystadenomas were negative. All mucinous cystic tumours of the pancreas are regarded potentially malignant (Compagno & Oertel, 1978). It is possible that the inability to produce TATI correlates with the degree of malignancy of mucinous cystic tumours.

As a conclusion, a negative TATI level, especially in urine, strongly speaks against pancreatic cancer, but in clinical practice the low specificity limits the use of TATI as a tumour marker to differentiate between benign and malignant pancreatic diseases.

This study has been supported by grants from Finska Läkaresällskapet, the Finnish Cancer Society, Svenska Kulturfonden and the Oskar Öflund Foundation, the Sigrid Juselius Foundation, the Finnish Academy and the Finnish Life Insurance Association.

### References

BARTELT, D., SHAPANKA, R. & GREENE, L. (1977). The primary structure of the human pancreatic secretory trypsin inhibitor. Amino acid sequence of the reduced S-aminoethylated protein. *Arch. Biochem. Biophys.*, **179**, 189.

COMPAGNO, J. & OERTEL, J. (1978). Mucinous cystic neoplasms of the pancreas with overt and latent malignancy (cystadenocarcinoma and cystadenoma). A clinicopathological study of 41 cases. *Am. J. Clin. Pathol.*, **69**, 573.

EDDELAND, A. & OHLSSON, K. (1978). A radioimmunoassay for measurement of human pancreatic secretory trypsin inhibitor in different body fluids. *Hoppe-Seyler's Z. Physiol. Chem.*, **359**, 671.

HAGLUND, C., ROBERTS, P.J., KUUSELA, P., SCHEININ, T.M., MÄKELÄ, O. & JALANKO, H. (1986). Evaluation of CA 19-9 as a serum tumour marker in pancreatic cancer. *Br. J. Cancer*, **53**, 197.

HALILA, H., HUHTALA, M.-L., SCHRÖDER, T., KIVILUOTO, T. & STENMAN, U.-H. (1985). Pancreatic secretory trypsin inhibitor-like immunoreactivity in pancreatectomized patients. *Clin. Chim. Acta*, **153**, 209.

HUHTALA, M.-L., PESONEN, K., KALKKINEN, N. & STENMAN, U.-H. (1982). Purification and characterization of a tumor-associated trypsin inhibitor (TATI) in urine of patients with gynecological malignancy. *J. Biol. Chem.*, **257**, 13713.

HUHTALA, M.-L., KAHANPÄA, K., SEPPÄLÄ, M., HALILA, H. & STENMAN, U.-H. (1983). Excretion of a tumor-associated trypsin inhibitor (TATI) in urine of patients with gynecological malignancy. *Int. J. Cancer*, **31**, 711.

KAZAL, L., SPICER, D. & BRABINSKY, R. (1948). Isolation of a crystalline trypsin inhibitor-anticoagulant protein from pancreas. *J. Amer. Chem. Soc.*, **70**, 3034.

KITAHARA, T., TAKATSUKA, Y., FUJIMOTO, K.-I., TANAKA, S., OGAWA, M. & KOSAKI, G. (1980). Radioimmunoassay for human pancreatic secretory trypsin inhibitor: Measurement of serum pancreatic trypsin inhibitor in normal subjects with pancreatic diseases. *Clin. Chim. Acta*, **103**, 135.

MARKS, W., OHLSSON, K. & POLLING, Å. (1984). Immunocytochemical distribution of trypsinogen and pancreatic secretory trypsin inhibitor in normal and neoplastic tissue in man. *Scand. J. Gastroenterol.*, **19**, 673.

MATSUDA, K., OGAWA, M., MURATA, A., KITAHARA, T. & KOSAKI, G. (1983). Elevation of serum immunoreactive pancreatic secretory trypsin inhibitor contents in various malignant diseases. *Res. Commun. Chem. Path. Pharm.*, **40**, 301.

MURATA, A., OGAWA, M., MATSUDA, K., MATSUURA, N. & KOSAKA, G. (1983). Immunoreactive pancreatic secretory trypsin inhibitor in gynecological diseases. *Res. Commun. Chem. Path. Pharm.*, **41**, 493.

OGAWA, M., KITAHARA, T., FUJIMOTO, K., TANAKA, S., TAKATSUKA, Y. & KOSAKI, G. (1980). Serum pancreatic secretory trypsin inhibitor in acute pancreatitis. *Lancet*, **ii**, 205.

STENMAN, U.-H., HUHTALA, M.-L., KOISTINEN, R. & SEPPÄLÄ, M. (1982). Immunochemical demonstration of an ovarian cancer-associated urinary peptide. *Int. J. Cancer*, **30**, 53.